# Perioperative Nerve Blockade Reduces Acute Postoperative Pain after Orthognathic Surgery

**DOI:** 10.1155/2023/7306133

**Published:** 2023-12-19

**Authors:** Yuka Oono, Saori Takagi, Lars Arendt-Nielsen, Hikaru Kohase

**Affiliations:** ^1^Division of Dental Anesthesiology, Department of Diagnostic and Therapeutic Sciences, Meikai University School of Dentistry, Sakado, Japan; ^2^Center for Neuroplasticity and Pain, SMI, Department of Health Science and Technology, School of Medicine, Aalborg University, Aalborg, Denmark; ^3^Department of Gastroenterology and Hepatology, Mech-Sense, Aalborg University Hospital, Aalborg, Denmark; ^4^Steno Diabetes Center North Denmark, Clinical Institute, Aalborg University Hospital, Aalborg, Denmark

## Abstract

**Background:**

The role of perioperative pain management is not only to reduce acute postoperative pain (POP) but also to prevent chronic POP. It would be important to know the usefulness of nerve blockade for perioperative management. However, it has not been extensively studied in orofacial surgery. The objective of the study was to investigate whether perioperative nerve blockade reduces acute POP after orthognathic surgery.

**Methods:**

Patients scheduled for orthognathic surgery were retrospectively reviewed (“preblock group”: the nerve blockade was performed before emergence from general anesthesia, and “no preblock group”: the nerve blockade was not performed before emergence from general anesthesia). The visual analog scale (VAS; 0–100 mm)-POP intensity, the VAS-POP areas under the curves (VASAUCs (mm × day)) in addition to VASAUCs for postoperative hours 6 (VASAUC_6), 12 (VASAUC_12), 18 (VASAUC_18), and 24 (VASAUC_24), the analgesic requirement period (day), and the number of days with pain (day) were analyzed. Data are presented as median (interquartile range) values.

**Results:**

Fifty-six patients (preblock group, 22; no preblock group, 34) were included (21 males, 35 females; age: 22.0 [21.0–28.0] years). VASAUC_6, VASAUC_12, VASAUC_18, and VASAUC_24 in the preblock group were significantly smaller than those in the no preblock group (3.5 [2.0–7.2] vs. 7.4 [5.1–10.0], *p* = 0.0007; 9.5 [6.4–13.7] vs. 15.0 [7.2–22.9], *p* = 0.042; 15.7 [10.3–23.1] vs. 29.3 [18.9–37.2], *p* = 0.0002; and 17.6 [12.7–27.2] vs. 39.5 [22.9–46.9], *p* = 0.001, respectively). There were no significant differences between the 2 groups in VASAUC, the analgesic requirement period, and the number of days with pain (*p* > 0.05).

**Conclusions:**

Perioperative nerve blockade reduces POP after orthognathic surgery, especially for the acute postoperative period.

## 1. Introduction

The role of perioperative pain management is not only to reduce acute postoperative pain (POP) but also to prevent the development of chronic POP [1, 2]. Acute pain management/acute pain service (APS) is being increasingly established to provide good POP control and hence reduce analgesic consumption [3–5]. Acute POP is an important clinical issue as it can have significant effects on rehabilitation, length of hospital stay and/or hospital readmission, and adverse events related to excessive analgesic use [6]. Poorly controlled acute POP has also been suggested as a risk factor for the development of chronic POP [7, 8], and the use of multimodal analgesia for the management of acute POP is considered an important paradigm in the context of the development of chronic POP [9–12]. Thus, acute pain management/APS is of crucial importance in any surgical setting [4, 5, 13, 14], including in orofacial surgery, an area that has not been extensively studied [15]. APS using nerve blockade is useful for perioperative management [16], and it would be important to know whether this is also applicable in orthognathic surgery settings. We hypothesized that the perioperative nerve blockade would reduce acute postoperative pain after orthognathic surgery.

The aim of this study was to investigate the effect of nerve blockade for acute POP relief after orthognathic surgery.

## 2. Methods

### 2.1. Participants

This study was conducted at the Division of Dental Anesthesiology, Department of Diagnostic and Therapeutic Sciences, Meikai University School of Dentistry, Sakado, Japan. A retrospective review of patients scheduled for Le Fort type I osteotomy (Le Fort 1) and bilateral sagittal split ramus osteotomy (SSRO) between July 2017 and June 2023 was performed.

The study was conducted in accordance with the guidelines of the Declaration of Helsinki and the World Medical Association and was approved by the Ethics Committee of Meikai University (approval number: A1803, approval date: June 15, 2018). The requirement for informed consent was waived due to the retrospective nature of the study. Study information was available on the website (https://www.meikai.ac.jp/dent/kenkyubu.html), and participants could opt out at any point.

### 2.2. Sample Size Calculation

An *a priori* power analysis was performed to establish the necessary sample size for this study using G^*∗*^Power (version 3.1.9.7) [17] with a probability of type I error of 0.05, a power of 0.8, and an effect size of 0.8. Based on these parameters, the power analysis demonstrated that a total sample size of 52 was required for this study. Since some data may be missed due to various reasons, the initial sample size was increased by 10%. Therefore, the final sample size for this study was 56.

### 2.3. Anesthesia

Orthognathic surgeries were performed under balanced general anesthesia with endotracheal intubation using propofol or remimazolam besylate, remifentanil hydrochloride, and rocuronium bromide. Infiltration anesthesia using 1% lidocaine hydrochloride monohydrate with epinephrine (1 : 100,000) was performed 5 min before surgery by the surgeon. Acetaminophen (2000 mg if the body weight of the patient was over 50 kg or 1000 mg if the body weight of the patient was under 50 kg) was administered 30 min before the end of the surgery. The dose of remifentanil and anesthetic agents was modified by the anesthesiologist based on the surgical stress and hemodynamic changes. A controlled arterial hypotension strategy with nitroglycerin was used.

### 2.4. Postoperative Pain Management and Evaluation of Postoperative Surgical Pain

For POP management, multimodal analgesia with nerve blockade was performed. The nerve blockade was performed by the anatomical landmark techniques with computed tomography evaluation using 0.5% levobupivacaine hydrochloride. The nerve blockade was performed at the anesthesiologist's discretion based on operative invasion, expected POP intensity, which was over 50/100 on the visual analog scale (VAS, 0–100 mm; the left endpoint (0) of the VAS indicated “no pain,” whereas the right endpoint (100) indicated the “worst pain imaginable”), or patient complaint. Acetaminophen, flurbiprofen axetil, loxoprofen sodium hydrate, or other pain medications including rescue analgesic administration were provided to individual patients for the management of POP. The prescription was decided at the anesthesiologist's discretion based on POP intensity and patients' general condition such as liver dysfunction or allergy.

Retrospectively, patients were divided into two groups—the “preblock group”: the nerve blockade was performed before emergence from general anesthesia, and the “no preblock group”: the nerve blockade was not performed before emergence from general anesthesia. In the no preblock group, if POP intensity on the VAS was over 50/100 and the patient requested the nerve blockade, the nerve blockade was performed.

An APS team consisting of anesthesiologists, nurses, and pharmacologists, in addition to surgeons, was established for POP management. The VAS was used to evaluate POP intensity (VAS-POP). On the operative day, VAS-POP assessments were performed by anesthesiologists at return to the ward (VAS_evaluation1_), 1 h after the patient returned to the ward (VAS_evaluation2_) at 7 pm and 9 pm, and if needed. From postoperative day (POD) 1 until discharge, VAS-POP was assessed at 8 am by anesthesiologists (during morning rounds) and at 6 am, 1 pm, and 7 pm by nurses. VAS-POP was also evaluated before analgesic administration.

Administration of scheduled analgesics was discontinued if VAS-POP was <30/100 or the patient did not request an additional prescription; this decision was made by anesthesiologists during the morning rounds. If VAS-POP did not reach 0/100 at discharge, patients were requested to record it until it reached 0/100. If the patient had POP after discharge, continuous analgesic treatment (acetaminophen or loxoprofen sodium hydrate) was performed in both the groups.

The period of analgesic administration was also recorded, and the postoperative “analgesic requirement period” was defined as “the final analgesic administration time (day)-the end time of anesthesia (day).” The number of days until VAS-POP reached 0/100 was designated as the “number of days with pain.” The VAS-POP areas under the curves (VASAUCs (mm × day)) were calculated by adding the corresponding VAS-POP areas. In addition, VASAUC for postoperative hours 6, 12, 18, and 24 was calculated and defined as VASAUC_6, VASAUC_12, VASAUC_18, and VASAUC_24 (mm × day), respectively.

The primary endpoint was VASAUC in addition to VASAUC_6, VASAUC_12, VASAUC_18, and VASAUC_24 ((mm × day)). Secondary outcome measures were VAS_evaluation1_ (mm), VAS_evaluation2_ (mm), the analgesic requirement period (days), and the number of days with pain (days). These parameters for the preblock group and the no preblock group were used for further analysis.

### 2.5. Statistical Analyses

The one-sample Kolmogorov–Smirnov test and the *F*-test for homogeneity of variance were performed before the *t*-tests. Depending on the results, the *t*-test, Welch *t*-test, or Mann–Whitney *U* test were performed to determine the difference between the two groups (preblock group (*n* = 22); no preblock group (*n* = 34)) for the demographic data (patient characteristics: age, height, body weight, and BMI and intraoperative variables: duration of surgery, duration of anesthesia, blood loss, 1% lidocaine for infiltration anesthesia, and remifentanil consumption) and postoperative pain parameters (VAS_evaluation1_, VAS_evaluation2_, VASAUC_6, VASAUC_12, VASAUC_18, VASAUC_24, VASAUC, analgesic requirement period (days), and number of days with pain (days)). Data are presented in terms of the median (interquartile range) values. All statistical analyses were performed using EZR (version 1.53; Jichi Medical University, Tochigi, Japan) [18]. Statistical significance was set at *p* < 0.05.

## 3. Results

### 3.1. Patient Characteristics and Intraoperative Variables

Patients scheduled for Le Fort 1 and bilateral SSRO between July 2017 and June 2023 was 60. Four patients were excluded after reviewing their medical records because of missing data. Finally, fifty-six patients were included in this study (21 males, 35 females; age: 22.0 [21.0–28.0] years). The number of patients in the preblock and no preblock groups was 22 and 34, respectively. Genioplasty was performed in addition to Le Fort type I osteotomy and bilateral SSRO for one patient in the preblock group (1/22; 4.5%) and three patients in the no preblock group (3/34; 8.8%). Patient demographic data (patient characteristics, surgery, and intraoperative variables) are summarized in Table 1.

There were no significant differences (*p* > 0.05) in the homogeneity of variance and normality for the demographic data including height, body weight, and BMI, duration of surgery, duration of anesthesia, blood loss, 1% lidocaine for infiltration anesthesia, and remifentanil consumption. Thus, *t*-tests were performed to determine the difference between the two groups (preblock group (*n* = 22); no preblock group (*n* = 34)) for height, body weight, BMI, duration of surgery, duration of anesthesia, blood loss, 1% lidocaine for infiltration anesthesia, and remifentanil consumption. There was a significant difference (*p* < 0.05) in the normality for the age in the no preblock group. Thus, the Mann–Whitney *U* test was performed to determine the difference between these two groups (preblock group (*n* = 22); no preblock group (*n* = 34)) for age.

There were no significant differences between the two groups' patient characteristics: age, height, body weight, and BMI (*p* > 0.05). Statistical tests showed that there were significant differences between the two groups for intraoperative variables: duration of surgery, duration of anesthesia, blood loss, 1% lidocaine for infiltration anesthesia, and remifentanil consumption (*p* < 0.05).

The analgesic administration in general anesthesia was as follows: In the preblock group, 2000 mg of acetaminophen was administered to 20 patients (20/22; 90.9%), whereas 1000 mg of acetaminophen was administered to 1 patient (1/22; 4.5%) whose body weight was under 50 kg; 100 mg of flurbiprofen axetil was administered to 1 patient (1/22; 4.5%), due to an acetaminophen allergy, and 50 mg of flurbiprofen axetil was administered to 3 patients (3/22; 13.6%). Fentanyl was not administered in the preblock group. In the no preblock group, 2000 mg of acetaminophen was administered to 31 patients (31/34; 91.2%), whereas 1000 mg of acetaminophen was administered to 3 patients (3/34; 8.8%) whose body weight was under 50 kg; 100 mg of flurbiprofen axetil was administered to 1 patient (1/34; 2.9%) who was administered 1000 mg of acetaminophen, and 50 mg of flurbiprofen axetil was administered to 1 patient (1/34; 2.9%). 100 *μ*g of fentanyl was administered to 2 patients (2/34; 5.9%) after extubation because of severe pain and hyperventilation.

### 3.2. Nerve Blockade and POP Management within 1 Hour Postoperatively

In the preblock group, the bilateral maxillary nerve block and the bilateral mandibular nerve block were performed in all patients (*N* = 22) after surgery and before emergence from general anesthesia by the anesthesiologist. The administered dose of the agent for the nerve blockade was 14.0 [10.3–16.0] mL of 0.5% levobupivacaine hydrochloride. Concerning one patient who underwent genioplasty in addition to Le Fort type I osteotomy and bilateral SSRO, a bilateral inferior alveolar nerve block was performed in addition to the bilateral maxillary nerve block and the bilateral mandibular nerve block. Seven patients (7/22; 31.8%) needed additional analgesic administration (50 mg of flurbiprofen axetil (*N* = 7)) back at the ward.

In the no preblock group (*N* = 34), 16 patients (16/34; 47.0%) underwent the nerve blockade before or after patient returned to the ward (Table 2). The details of the nerve block were as follows: right maxillary nerve block (*N* = 4), left maxillary nerve block (*N* = 3), right mandibular nerve block (*N* = 5), left mandibular nerve block (*N* = 4), right inferior alveolar nerve block (*N* = 5), left inferior alveolar nerve block (*N* = 3), and infiltration anesthesia (*N* = 5). The administered dose of the agent for the nerve blockade was 10.0 [7.5–10.0] mL of 0.5% levobupivacaine hydrochloride. Nine patients (9/34; 26.5%) underwent analgesic administration: 1000 mg of acetaminophen (*N* = 4), 50 mg of flurbiprofen axetil (*N* = 3), 1000 mg of acetaminophen and 50 mg of flurbiprofen axetil (*N* = 1), and 50 mg of indomethacin (*N* = 1). Nine patients (9/34; 26.5%) did not need additional analgesia.

### 3.3. Postoperative Analgesic Consumption

In the preblock group, 21 patients without the acetaminophen allergy patient (21/22; 95.5%) were prescribed acetaminophen. The median total dose of acetaminophen was 4,750 [2,000–17,625] mg. Concerning other analgesics, 17 patients (17/22; 77.3%) required the administration of flurbiprofen axetil. The median total dose of flurbiprofen axetil was 100 [50–100] mg. Nineteen patients (19/22; 86.4%) required the administration of loxoprofen sodium hydrate. The total dose of loxoprofen sodium hydrate was 510 [255–840] mg, and 15 mg of pentazocine was administered to one patient on the operative day.

In the no preblock group, all patients (34/34; 100%) were prescribed acetaminophen. The median total dose of acetaminophen was 20,500 [15,000–24,000] mg. Concerning other analgesics, 10 patients (10/34; 29.4%) required the administration of flurbiprofen axetil: 50 mg of flurbiprofen axetil was administered to 8 patients, and 100 mg of flurbiprofen axetil was administered to 2 patients. Twenty-seven patients (27/34; 79.4%) required the administration of loxoprofen sodium hydrate. The total dose of loxoprofen sodium hydrate was 480 [180–885] mg. Eight patients required indomethacin: while 50 mg of indomethacin was administered to 7 patients, 100 mg of indomethacin was administered to 1 patient.

### 3.4. VASAUCs, the Analgesic Requirement Period, and the Number of Days with Pain

**Figure 1:**
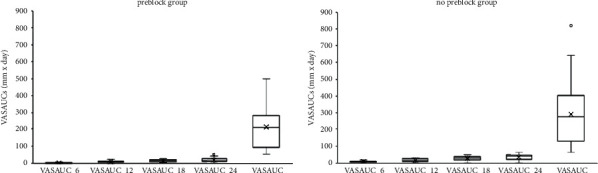
Boxplots for VASAUCs for the preblock group and the no preblock group. VAS, visual analog scale; VASAUC, VAS score area under the curve; VASAUC_6, VASAUC for postoperative hour 6; VASAUC_12, VASAUC for postoperative hour 12; VASAUC_18, VASAUC for postoperative hour 18; VASAUC_24, VASAUC for postoperative hour 24.

**Figure 2:**
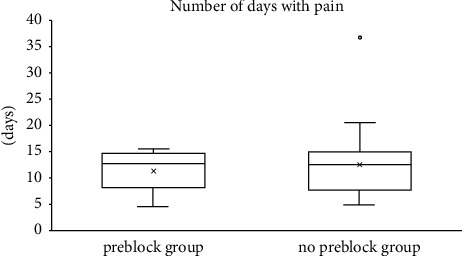
Boxplots for the number of days with pain for the preblock group and the no preblock group.

VASAUCs, the analgesic requirement period, and the number of days with pain are shown in Table 3 and Figures 1 and 2.

There were no significant differences (*p* > 0.05) in the homogeneity of variance and normality for VAS_evaluation1_, VAS_evaluation2_, VASAUC_6, VASAUC_12, VASAUC_18, VASAUC_24, and VASAUC. Thus, *t*-tests were performed to determine the difference between the two groups (preblock group (*n* = 22); no preblock group (*n* = 34)) for VAS_evaluation1_, VAS_evaluation2_, VASAUC_6, VASAUC_12, VASAUC_18, VASAUC_24, and VASAUC. The *t*-test showed that VAS_evaluation1_, VAS_evaluation2_, VASAUC_6, VASAUC_12, VASAUC_18, and VASAUC_24 in the preblock group were significantly lower than those in the no preblock group (*p* = 0.001, 0.034, 0.0007, 0.042, 0.0002, and 0.001, respectively). There was no significant difference in VASAUC (*p* = 0.093) between the groups.

There was a significant difference (*p* < 0.05) in the homogeneity of variance for the analgesic requirement period and the number of days with pain. Thus, the Welch *t*-test was performed to determine the difference between the two groups (preblock group (*n* = 22); no preblock group (*n* = 34)) for the analgesic requirement period and the number of days with pain. The test showed that there were no significant differences in the analgesic requirement period and the number of days with pain between the 2 groups (*p* = 0.215 and *p* = 0.422, respectively).

## 4. Discussion

This is the first study to show that the nerve blockade before emergence from general anesthesia can reduce acute POP at back to the ward and 1 h after back to the ward, in addition to 6 h, 12 h, 18 h, and 24 h, compared to the no nerve blockade before emergence from general anesthesia, indicating that the nerve blockade before emergence from general anesthesia results in good quality pain management. These results demonstrate that the nerve blockade before emergence from general anesthesia with APS reduces postoperative pain, especially for the acute postoperative period, and is effective for providing POP relief in an orthognathic surgery.

### 4.1. POP after Orthognathic Surgery and Perioperative Nerve Blockade

APSs are being increasingly established to provide good control of POP and hence reduce analgesic consumption [3–5]. However, the quality of POP therapy can vary greatly between different departments [14]. Recently, guidelines for acute pain management have been published [14], but initiatives specific to the orofacial field are lacking; only one article on different options available to dental clinicians has been published concerning nerve blockade [15].

Multimodal analgesic intervention (including acetaminophen and NSAID administration) [16] should include several drugs and techniques to combat peripheral and central neuroplasticity [3]. Well-known pharmacologic agents used for pain control include acetaminophen, ibuprofen, anticonvulsants, opioids, gamma-aminobutyric acid agonists, and local anesthetics [15]. Combined analgesic regimens are more effective in achieving POP control and avoiding untoward side effects than single-drug regimens [15]. In an attempt to minimize the risk of the transition from acute to chronic pain, regional analgesia has been recommended [19]. The importance and effectiveness of the nerve blockade in oral surgery have also been reported [20–22], and peripheral nerve blocks are increasingly being used as a component of multimodal analgesia [23].

A recent randomized controlled clinical trial showed that postoperative bupivacaine use did not show superior analgesic efficacy when compared with normal saline in controlling acute POP after bilateral SSRO [24]. The duration of action for 0.5% levobupivacaine hydrochloride is approximately 6 h [25], and the elimination half-life for bupivacaine hydrochloride hydrate is also approximately 6 h [26]. Once the analgesic effects subside, pain reoccurs. Thus, efficient pain management by APS is important to prevent the onset of sudden, severe POP after the effect of a block wears off. The current study demonstrated that APS with a perioperative nerve blockade is useful for the management of POP. For successful pain control using the nerve blockade, it is important that the APS team continuously evaluates patient POP intensity based on medical interviews to determine the appropriate multimodal analgesia procedures, such as blockade, rescue analgesic administration, and scheduled analgesic administration, for individual patients.

Control of acute and subacute POP is useful for reducing the onset of chronic POP [9–12], and the intensity and duration of acute POP pain have often been found to be major risk factors for developing chronic POP [7, 8, 10, 27]. An observational study reported that one of the risk factors for chronic POP is the amount of time spent in severe unrelieved pain on the first postoperative day [7]. Considerable attention has therefore been focused on controlling acute POP as a possible preventive strategy [27]. In addition, the need for continuous analgesic treatment after discharge is emphasized after total hip and knee arthroplasty to ensure postoperative function and facilitate rehabilitation [28]. Appropriate postoperative follow-up after discharge may lead to an appropriate preventive strategy for chronic POP [4].

### 4.2. Clinical Implication

The intraoperative variables were significantly different in the two groups; the preblock group showed longer duration of surgery and more blood loss. The nerve blockade was performed at the anesthesiologist's discretion based on operative invasion and expected POP intensity. This would result in the significant difference in the two groups. Despite bigger surgical stress (longer duration of surgery and more blood loss) in the preblock group, the postoperative pain parameters evaluated as VAS-POP and VASAUC in the acute postoperative period (24 h) resulted in less POP compared to the no preblock group. These results are important clinical implications as control of acute and subacute POP, especially on the first postoperative day [7], is useful for reducing the onset of chronic POP [9–12].

### 4.3. Limitations

A limitation of this study was that it was retrospective but not randomized. Therefore, the results could be affected by clinical factors such as patients' pain. Regardless, the current study clearly demonstrated that the nerve blockade before emergence from general anesthesia and management with APS is effective for POP relief after orthognathic surgery.

## 5. Conclusion

The nerve blockade before emergence from general anesthesia and management with APS was found to be effective for POP relief after orthognathic surgery.

APS with a perioperative nerve blockade in combination with suitable multimodal analgesia management should be considered for effective control of acute and subacute POP. Moreover, the nerve blockade with APS could result in effective prevention of the onset of chronic POP in the future.

## Figures and Tables

**Table 1:** Patient characteristics, surgery, and intraoperative variables.

	Preblock group	No preblock group	*p* value
Patient characteristics			
Sex (M/F), *n* (%)	8 (36.4)/14 (63.6)	13 (38.2)/21 (61.8)	—
Age (years)	22.0 [21.0–28.0]	22.5 [21.0–26.8]	0.866
Height (cm)	163.2 [158.0–171.6]	165.6 [159.4–171.0]	0.529
Body weight (kg)	56.0 [51.9–62.8]	57.0 [52.3–59.9]	0.525
BMI	21.2 [19.4–22.6]	20.6 [19.1–22.0]	0.219
ASA physical status (1/2/3), *n* (%)	15 (68.2)/6 (27.3)/1 (4.5)	26 (76.5)/8 (23.5)	—
Surgery			
Le Fort type I osteotomy (Le Fort 1) and bilateral sagittal split ramus osteotomy (SSRO)	21/22; 95.5%	31/34; 91.2%	—
Le Fort type I osteotomy (Le Fort 1), bilateral sagittal split ramus osteotomy (SSRO), and genioplasty	1/22; 4.5%	3/34; 8.8%	—
Intraoperative variables			
Duration of surgery (min)	368.0 [349.3–440.3]	311.5 [261.0–365.3]	0.001^*∗∗*^
Duration of anesthesia (min)	495.0 [473.5–560.8]	423.5 [377.5–483.3]	0.0007^*∗∗*^
Blood loss (ml)	735.5 [559.0–962.0]	564.5 [377.0–703.0]	0.042^*∗*^
1% lidocaine for infiltration anesthesia (ml)	25.0 [25.0–27.3]	20.0 [17.6–25.0]	0.003^*∗∗*^
Remifentanil consumption (mg)	10.97 [8.75–13.15]	7.65 [6.33–9.25]	0.004^*∗∗*^

Preblock group, nerve blockade was performed before emergence from general anesthesia; no preblock group, nerve blockade was not performed before emergence from general anesthesia. F, female; M, male; BMI, body mass index; *n*, number of patients. Preblock group, *N* = 22; no preblock group, *N* = 34. Data are presented as numbers or median (interquartile range) values. *T*-tests were performed to compare 2 groups in spite of age which was performed with the Mann–Whitney *U* test. ^*∗*^*p* < 0.05; ^*∗∗*^*p* < 0.005.

**Table 2:** Additional intervention for the patients in the no preblock group.

Patient number	Type of intervention	The administered dose of 0.5% levobupivacaine hydrochloride for nerve blockade (ml)	Postoperative timing of the execution
1	Infiltration anesthesia	6	At the ward
2	Infiltration anesthesia	10	At the ward
3	Right inferior alveolar nerve block	5	At the ward
4	Right inferior alveolar nerve block, right mandibular nerve block	5	At the ward
5	Right mandibular nerve block, left mandibular nerve block	9.5	At the ward
6	Right mandibular nerve block, left mandibular nerve block	8	At the ward
7	Right inferior alveolar nerve block, left inferior alveolar nerve block	10	At the ward
8	Right mandibular nerve block, left mandibular nerve block	8	At the ward
9	Right inferior alveolar nerve block, left inferior alveolar nerve block	10	At the ward
10	Infiltration anesthesia	10	At the ward
11	Right mandibular nerve block, left mandibular nerve block	10	At the ward
12	Infiltration anesthesia	10	At the ward
13	Right maxillary nerve block	4	Before back to the ward (in the operation room)
14	Right inferior alveolar nerve block, left inferior alveolar nerve block, right maxillary nerve block, left maxillary nerve block	25	Before back to the ward (in the operation room) and at the ward
15	Right maxillary nerve block, left maxillary nerve block	10	Before back to the ward (in the operation room)
16	Infiltration anesthesia, right maxillary nerve block, left maxillary nerve block	10	Before back to the ward (in the operation room)

**Table 3:** Postoperative pain parameters.

	Preblock group	No preblock group	*p* value
VAS_evaluation1_	12.5 [0.0–50.0]	50.0 [31.3–77.5]	0.001^*∗∗*^
VAS_evaluation2_	17.5 [4.0–30.0]	30.0 [16.3–45.3]	0.034^*∗*^
VASAUC_6 (mm × day)	3.5 [2.0–7.2]	7.4 [5.1–10.0]	0.0007^*∗∗*^
VASAUC_12 (mm × day)	9.5 [6.4–13.7]	15.0 [7.6–22.9]	0.042^*∗*^
VASAUC_18 (mm × day)	15.7 [10.3–23.1]	29.3 [18.9–37.2]	0.0002^*∗∗*^
VASAUC_24 (mm × day)	17.6 [12.7–27.2]	39.5 [22.9–46.9]	0.001^*∗∗*^
VASAUC (mm × day)	214.4 [96.2–263.1]	273.3 [141.8–399.7]	0.093
Analgesic requirement period (days)	7.2 [6.1–10.0]	8.1 [6.1–10.2]	0.215
Number of days with pain (days)	12.7 [8.7–14.5]	12.6 [7.9–14.4]	0.422

Preblock group, nerve blockade was performed before emergence from general anesthesia; no preblock group, nerve blockade was not performed before emergence from general anesthesia. VAS, visual analog scale; VASAUC, VAS score area under the curve; VASAUC_6, VASAUC for postoperative hour 6; VASAUC_12, VASAUC for postoperative hour 12; VASAUC_18, VASAUC for postoperative hour 18; VASAUC_24, VASAUC for postoperative hour 24; VAS_evaluation1_ denotes the operative pain intensity at return to the ward. VAS_evaluation2_ denotes the operative pain intensity 1 hour after the patient returned to the ward. The number of days with pain denotes the number of days required for postoperative pain to reach 0/100 on the VAS. *N*, number of patients. Preblock group, *N* = 22; no preblock group, *N* = 34. Data are presented as median (interquartile range) values. The *t*-test was performed to determine the difference between two groups in spite of the analgesic requirement period and number of days with pain, which was performed with the Welch *t*-test. ^*∗*^*p* < 0.05; ^*∗∗*^*p* < 0.005.

## Data Availability

The data that support the findings of this study are available from the corresponding author upon reasonable request.
